# ChatGPT for Tinnitus Information and Support: Response Accuracy and Retest after Three and Six Months

**DOI:** 10.3390/brainsci14050465

**Published:** 2024-05-07

**Authors:** W. Wiktor Jedrzejczak, Piotr H. Skarzynski, Danuta Raj-Koziak, Milaine Dominici Sanfins, Stavros Hatzopoulos, Krzysztof Kochanek

**Affiliations:** 1 Department of Experimental Audiology, World Hearing Center, Institute of Physiology and Pathology of Hearing, 05-830 Kajetany, Poland; k.kochanek@ifps.org.pl; 2 Department of Teleaudiology and Screening, World Hearing Center, Institute of Physiology and Pathology of Hearing, 05-830 Kajetany, Poland; p.skarzynski@csim.pl (P.H.S.); msanfins@uol.com.br (M.D.S.); 3 Institute of Sensory Organs, 05-830 Kajetany, Poland; 4 Heart Failure and Cardiac Rehabilitation Department, Faculty of Medicine, Medical University of Warsaw, 03-242 Warsaw, Poland; 5 Tinnitus Department, World Hearing Center, Institute of Physiology and Pathology of Hearing, 05-830 Kajetany, Poland; d.koziak@ifps.org.pl; 6 Speech-Hearing-Language Department, Audiology Discipline, Universidade Federal de São Paulo, São Paulo 04023062, Brazil; 7 ENT and Audiology Unit, Department of Neurosciences and Rehabilitation, University of Ferrara, 44121 Ferrara, Italy; sdh1@unife.it

**Keywords:** chatbot, large language model, natural language processing, artificial intelligence, self-diagnosis, hearing, audiology, longitudinal, otorhinolaryngology

## Abstract

Testing of ChatGPT has recently been performed over a diverse range of topics. However, most of these assessments have been based on broad domains of knowledge. Here, we test ChatGPT’s knowledge of tinnitus, an important but specialized aspect of audiology and otolaryngology. Testing involved evaluating ChatGPT’s answers to a defined set of 10 questions on tinnitus. Furthermore, given the technology is advancing quickly, we re-evaluated the responses to the same 10 questions 3 and 6 months later. The accuracy of the responses was rated by 6 experts (the authors) using a Likert scale ranging from 1 to 5. Most of ChatGPT’s responses were rated as satisfactory or better. However, we did detect a few instances where the responses were not accurate and might be considered somewhat misleading. Over the first 3 months, the ratings generally improved, but there was no more significant improvement at 6 months. In our judgment, ChatGPT provided unexpectedly good responses, given that the questions were quite specific. Although no potentially harmful errors were identified, some mistakes could be seen as somewhat misleading. ChatGPT shows great potential if further developed by experts in specific areas, but for now, it is not yet ready for serious application.

## 1. Introduction

Chat generative pre-trained transformer (ChatGPT) by OpenAI is a conversational tool based on artificial intelligence. It has recently attracted a high level of interest [1]. ChatGPT is based on large language models (LLMs) and is capable of human-like conversation. It is now being tested in various domains of knowledge, including science and medicine. For example, it has been used to answer questions about national medical examinations [2,3], psychiatry [4], hemophilia [5], and colon cancer [6]. In the case of hearing, there are only a few studies that have used ChatGPT. It has been evaluated as a potential patient information source in otolaryngology [7] and has been used to assist with medical documentation in cases of Eustachian tube dysfunction [8].

In one article that has considered the future application of chatbots to hearing health care, Swanepoel and colleagues discussed its possible use by patients, clinicians, and researchers [9]. In the case of patients, Swanepoel and colleagues suggest that chatbots could be used for initial screening, making recommendations for interventions, education, support, and teleaudiology. Of course, before this becomes possible, chatbots would first need to be evaluated in terms of their accuracy relative to current best knowledge.

Here, we test the possible application of ChatGPT to tinnitus, an important medical and scientific topic within audiology and otolaryngology. The problem with tinnitus is that it is still not understood, especially in the case of subjective tinnitus, where the underlying pathophysiology is not known [10]. Moreover, there is no objective test [11], and there is no treatment that provides positive outcomes for the majority of sufferers [12]. At the same time, there are very many sufferers: one meta-analysis reported that around 10% of the global population has chronic tinnitus [13]. Recent studies have pointed to an apparent increase in hearing impairment as well as tinnitus, and further increases in these problems might be expected [14,15].

Kutyba and colleagues recently found that a large percentage of tinnitus sufferers actively seek solutions for themselves [16], and some studies indicate that tinnitus sufferers seek information and help on the Internet [17]. Furthermore, researchers from other disciplines are beginning to ask, “What if your patient switches from Dr. Google to Dr. ChatGPT?” [18]. The growing interest in using ChatGPT has been noted in recent press reports, which have been linked to a rise at the start of the school year [19]. Therefore, we presume that a significant number of people might turn to chatbots to learn more about their tinnitus. These individuals may include patients, their families, students, or even physicians.

There are several issues that may limit the performance of ChatGPT and provide a rationale for the current study. ChatGPT responses are generated based on training data, which consist of texts from the internet, books, articles, and other written materials curated and processed by its creators [20]. However, it cannot access real-time information or browse the web for updates. Additionally, there is no built-in filtering mechanism for medical information, although users can create prompts and ask for more specific or professional responses. It is not known if a chatbot has been updated since its release, and there seem to be no studies that have looked at changes in chatbot behavior over longer periods.

The purpose of this study was to evaluate ChatGPT in terms of the accuracy of its responses to a defined set of questions about tinnitus. Furthermore, given the technology is quickly advancing, we evaluated responses to the same questions after a further 3 and 6 months. An earlier version of this article was posted to the medRxiv preprint server on 19 December 2023.

## 2. Materials and Methods

We framed 10 questions related to tinnitus, questions which, in our opinion, are quite common (Table 1). The questions fell into two categories. The first 5 questions were fairly basic and were based on questions that we often hear from people experiencing tinnitus (or their relatives). A second set of 5 more specialized questions related to those a student of audiology or otolaryngology might ask or someone with a deeper knowledge of tinnitus, such as a physician or researcher. They were based on topics that are an important part of tinnitus research but might also be information related to a patient seeking a diagnosis or by a beginning audiologist, e.g., information on questionnaires used for tinnitus evaluation. These questions were based on information that could be verified against specific research papers: question 6 (e.g., [21]), question 7 (e.g., [22,23]), question 8 (e.g., [21,24]), question 9 (e.g., [25,26]), and question 10 (e.g., [27,28]). All questions were of the open type but were made deliberately diverse in order to explore the limits of ChatGPT. Some questions asked ChatGPT to answer just yes or no (questions 3, 5, and 8), while others required a longer answer. Some questions were very short and general (e.g., questions 2 and 4), and some were quite long and specific (e.g., questions 3 and 10).

We presented the questions to chatbot ChatGPT version 3.5 during three sessions, the first on 21 August 2023, the second—3 months later—on 26 November 2023, and the third—6 months later—on 1 March 2024. We used version 3.5 (not the paid version) since it is free and probably used by most people. The responses from each session were copied to a single file (Supplementary Files S1–S3). The set of responses was presented to 6 experts (the authors) with several years of experience in tinnitus research, documented by numerous publications. More specifically, two were medical doctors, two were biomedical engineers, and two were audiologists, but all were working in the field of audiology.

The standard of each response was rated by the experts on a 5-point Likert scale (1 = extremely unsatisfactory, 2 = unsatisfactory, 3 = neutral, 4 = satisfactory, and 5 = extremely satisfactory). Each of the experts evaluated the responses independently. A similar approach has been used in tests of ChatGPT in other disciplines [29,30]. Evaluations were undertaken within one week after the questions were presented to ChatGPT (at the first session, the experts were unaware of potential follow-up sessions). Experts were instructed to include in the scoring an assessment of the correctness and completeness of the answers, just as they would if they were grading a student on an exam.

Additionally, we counted the number of words in each response (using Microsoft Word, Microsoft Office Professional Plus 2013), recorded whether references were provided, and took note of whether consultation with a specialist was suggested.

### Statistical Analysis

Analyses were made in Matlab (version 2023b, MathWorks, Natick, MA, USA). Repeated measures analysis of variance (rmANOVA), chi-squared test, and t-test were used to assess differences. In all analyses, a 95% confidence level (*p* < 0.05) was taken as the criterion of significance. When multiple comparisons with *t*-tests were performed, *p*-values were adjusted using the Benjamini and Hochberg [31] procedure to control for false discovery rates.

## 3. Results

Table 2 presents the average scores given to each ChatGPT response by the six experts for the three sessions. For seven of the questions, the average rating of responses increased for the second and third sessions in comparison with session one. The differences between the second and third sessions were much smaller. One of the responses (response to question 3) scored a maximum of 5 in all three sessions. However, for individual questions, there was no statistically significant change over time.

Table 3 shows the overall average for all questions and the number of responses that were either satisfactory or extremely satisfactory. ANOVA showed that, over the three sessions, there was a statistically significant difference in average scores (ratings of responses to questions 1–10; last column of Table 3). Pairwise comparisons revealed that the average score increased significantly between the first and second sessions (*p* = 0.0093) and also between the first and third sessions (*p* = 0.010). ANOVA showed that, over the three sessions, there was a statistically significant difference in average scores for responses to basic questions, but pairwise comparisons did not confirm that (no significant differences between responses to basic questions at Sessions 1 and 2, 1 and 3, and 2 and 3).

It can be seen that most of the responses given by ChatGPT were rated as satisfactory or better in the first session, while all of them achieved this rating in the second and third sessions (Table 3). Nevertheless, there were a few particular instances where the responses were not perfectly true and might be considered slightly misleading (especially for the first session). This aspect is further explored in the Discussion.

The scores assigned by each expert to ChatGPT’s responses were compared to a mid-rating of 3.0 (i.e., a total score of 30 for 10 responses). Our expectation was that a competent set of answers should achieve a better rating than that. Despite some differences between the experts, all scores were significantly better than 3.0 for the first session as well as for the second and third (for all comparisons, *p* < 0.0001).

Table 4 presents some additional evaluations. All responses lacked references to sources of information, and when specifically asked for references, the tool generated artificial ones. This behavior is described in more detail in the discussion. Most responses in sessions 1 and 3 suggested consultation with a specialist, and in session 2, all the responses contained this suggestion (Table 4). Responses given by ChatGPT were quite long: on average, 431 words for the first session, 411 for the second, and 368 for the third (Table 4). The pairwise comparisons revealed that the number of words decreased significantly between the first and third (*p* = 0.0007) and second and third sessions (*p* = 0.0005).

## 4. Discussion

The majority of ChatGPT responses were rated as satisfactory or better. There were only a few scores that fell below this level. This shortfall occurred only in the first session, and it seems that the quality of responses improved significantly over the subsequent 3 months and remained quite stable over the following 3 months. We were surprised at ChatGPT’s general degree of competency since tinnitus is a complex topic that includes many difficult aspects. In our opinion, ChatGPT provided reasonable responses to basic questions. The answers were easy to understand. Indeed, they were typically clearer than the responses we received from most of our students. One important aspect, and one we rate positively, is that ChatGPT conveys the importance of professional assessment and care in cases of tinnitus. Furthermore, it emphasizes that treatment depends on the needs of the individual patient and, to be most effective, usually needs to employ multiple approaches.

At the same time, ChatGPT’s responses sometimes lacked a degree of focus. They often consisted of a list of topics related to the question but without any detailed analysis, clear connections, or deeper aspects of the problem. This reflects the fact that most of the responses were too long and contained extraneous information. Sometimes, ChatGPT appeared to add extra information just to reach a predetermined number of words. This observation parallels the result of another study that compared ChatGPT responses to responses by experts and concluded that, while ChatGPT responses were longer, their medical accuracy was lower than that of experts [32].

When we looked at actual responses, certain patterns of successes and failures began to emerge. This was especially the case in the first session (the reader can explore the responses themselves in Supplementary Files S1–S3). Starting with the positives, we draw attention to question 3, which was constructed specifically to address the problem of advertisements that promise quick remedies for tinnitus. These advertisements are commonly encountered on web pages and news portals or are sent by email. They are potentially harmful in that, as well as losing money, patients may put off contacting a specialist and delay proper treatment. ChatGPT’s response in this case is very balanced. It starts by saying, “It’s important to approach advertisements for medications that claim to treat tinnitus in a very short period with caution and skepticism”. It continues by giving more details about the complexity of tinnitus, the need for scientific support, and placebo effects (see Supplementary File S1), and finishes with a recommendation about seeking personalized advice from a healthcare professional. In our opinion, this response shows the great potential of chatbots to provide reasonable answers and recommendations about difficult and controversial issues. Other studies seem to back up this conclusion [33]; however, there are also reports showing that ChatGPT sometimes creates misinformation [29,34]. Nevertheless, the possibility of it being able to detect potentially unreliable information seems to suggest that some external moderation of the AI is taking place. Based on random information taken from the Internet, an AI program could easily recommend such treatments. At the same time, we are puzzled as to how this could be avoided, for it would seem very difficult, without being an expert, to pre-train ChatGPT to identify such treatments as suspicious.

We now move on to examples that show the sort of mistakes ChatGPT can make. In response to questions 1 and 2, ChatGPT said that reducing caffeine and salt intake may reduce tinnitus symptoms. In fact, although there are some studies pointing in this direction, the subject is controversial, and there is certainly no definite proof [35].

Question 7 asked about the relation of tinnitus to otoacoustic emissions (OAEs). Its reply referred only to evoked OAEs, even though OAEs can also be spontaneous [36], and these can be said to most closely resemble tinnitus. However, ChatGPT did not state that fact, restricting itself only to evoked OAEs and not giving any indication of different types of OAEs. There are some reports of spontaneous OAEs that have the same frequency as the subject’s tinnitus [22], and this is highly relevant. However, during session 1, the response only said, “For instance, individuals with tinnitus that is predominantly tonal might exhibit different OAE patterns compared to those with non-tonal tinnitus”. This is too general and needs further detail. Without additional information about the type of OAE (evoked or spontaneous), the measurement paradigm, and the relevant OAE parameters, this sentence is essentially useless. Moreover, the whole answer was quite long and convoluted, compounded by a lack of references to specific information. In sessions 2 and 3, there was only a small improvement in this aspect.

Question 8 related to a link between tinnitus and the psychological state of the patient. Although the response in sessions 1 and 2 was generally correct, it did not mention that such a link is usually present only for cases of severe chronic tinnitus, not for all instances of the condition [24]. However, in session 3 this was mentioned.

Question 9 asked about questionnaires used to study tinnitus. The response for session 1 mentioned a few popular questionnaires like the Tinnitus Handicap Inventory (THI) [25], the Tinnitus Functional Index (TFI) [26], and also, remarkably, a “Goebel and Hiller Questionnaire”. But we were unable to find such a thing in the literature. Goebel and Hiller did contribute to studies based on some questionnaires, but there seems to be no actual questionnaire named after them [37,38]. This seems to be a creation of ChatGPT, instances that are sometimes called ‘chatbot hallucinations’ [39]. This problem was not detected by us in the second and third sessions when a smaller number of questionnaires was also listed. On the other hand, there was no mention of some newer questionnaires that are gaining attention, and ChatGPT said nothing about the need to have questionnaires adapted to the patient’s native language [40,41].

**Figure 1:**
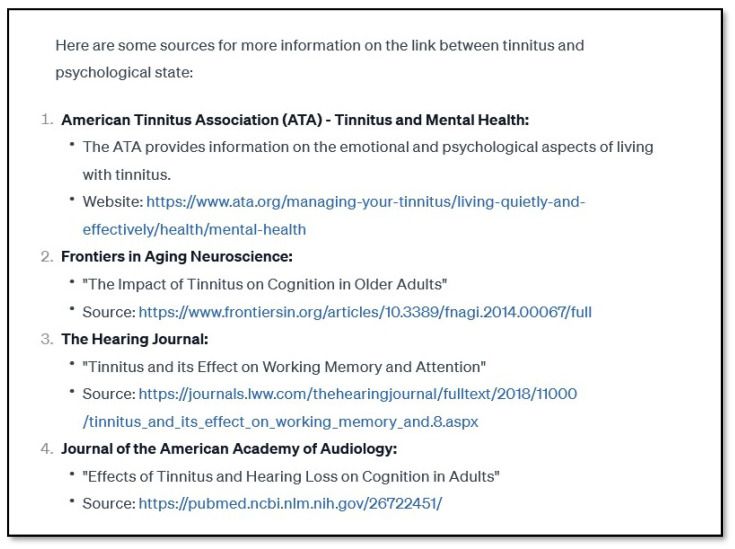
A screenshot of a portion of a ChatGPT response to question number 8 in which references were asked for. The prompt used was: “Is there a link between tinnitus and the psychological state of the patient? Provide sources of information”. Only the part of the response with references is shown.

The major drawback of ChatGPT is that, when not directly asked, it does not provide references. Such a lack may not be needed in casual conversation, but it is essential when discussing scientific or medical knowledge. When the phrase “with references” was added to each question, ChatGPT provided them during the first session, but they were totally artificial. False citations were created, with some plausible author names, a plausible title, and the name of some journal in which papers on the topic are published. This is similar to the creations or hallucinations mentioned in connection with the “Goebel and Hiller Questionnaire”. During the second session, we observed a change in that explicit links to references were often provided (see Figure 1). However, as we discovered, these, too, were artificial. The screenshot of a portion of the response to question 8 (Figure 1) shows four references. The first directs to a page that says: “Sorry! That page doesn’t seem to exist”. The second and fourth links to scientific papers on topics not related to tinnitus and with titles different from those ChatGPT gives. The third links to a paper related to tinnitus but with a title once again different from that in ChatGPT’s response. We did not observe any improvement in references between sessions 2 and 3. Some references pointed directly to webpages, with some being correct and some being ‘dead’ links. Occasionally, there were references to real scientific papers with all details correct. More commonly, the listed papers borrowed author names from one paper and merged them with the title of another paper from different authors; curiously, the result carried a real journal name but false page numbers and even a fake doi number.

The final limitation of ChatGPT in regard to providing sources of information is that it does not place references within the text but puts them all in a list underneath. That means one is unable to tell which part of the response they are referencing. In contrast, one other chatbot—Microsoft Copilot (formerly Bing Chat)—provides links to real sources and provides references in the text [42].

Often, in response to a specialized question, ChatGPT will provide a reasonable disclaimer, suggesting that a specialist should be consulted or that scientific knowledge is progressing and information might be out of date. A few times, it said that it was powered by knowledge up to September 2021. We view this aspect as positive since not many sources will suggest one should consult a specialist or that knowledge is limited (and even if it is suggested, it is usually only as part of a reference or as a disclaimer in small font). With ChatGPT, it was included as part of the whole response.

Some limitations of the study should be mentioned. In our study, we did not repeat the questions at any of the time points. Repeated trials at a fixed time would have helped statistically. As it stands, differences observed at different times might simply reflect randomness in the chatbot’s responses rather than any actual improvement. Nevertheless, we know that other studies have observed quite consistent ChatGPT responses when asked over short time frames (e.g., days) [43]. Another consideration is that the experts did not score all three sessions all at once but in sequence, so they might have expected to see better performance with time (although we did our best to be objective at each session). It is known that experts may give different ratings to the same responses when asked at different times. Also, we focused on ChatGPT version 3.5, while the more advanced ChatGPT 4 performs better in many disciplines [44,45]. Despite this, it could be assumed that most users will go for the earlier version since it is free.

This study has begun to explore issues such as the efficacy of AI tools in specialized medical fields and their long-term consistency. We hope that our findings will spark further discussion and inspire additional research. Future studies should broaden the range and complexity of tinnitus-related questions and incorporate patient evaluations so as to assess the comprehensiveness of the responses from their perspective. Moreover, extending the evaluation period beyond six months and implementing a feedback loop, where expert critiques directly inform AI training, could enhance AI performance. In the ChatGPT interface, it is possible to mark a response as a ‘bad response,’ which facilitates this process. While there are other freely available chatbots, such as Microsoft Copilot (formerly Bing Chat) and Google Gemini (formerly Bard), we selected ChatGPT as our test-bed due to its popularity and some studies suggesting its superior accuracy [42,46]. Nonetheless, to fully evaluate its utility, it is crucial to compare ChatGPT’s responses not only with those from other AI models but also against those generated by experts or traditional medical information resources.

## 5. Conclusions

Chatbots seem to be a tool that, theoretically, can be used in various areas of audiology, including tinnitus support. On our set of 10 questions, ChatGPT provided responses that exceeded our original expectations, especially given the fact that the questions were on a very specific topic. Although no potentially harmful errors were identified, there were some omissions that could be considered misleading. Scientifically, the big drawback is that no solid references were ever provided, and when specifically asked to provide references, they were, in most cases, artificial. ChatGPT shows considerable potential if developed by specialists in a particular area, but presently, it is not yet ready for serious applications. We did see some improvement in responses over the 6 months, but it is also possible they were random variations.

## Figures and Tables

**Table 1:** Questions used to test ChatGPT.

No	Question	
1	I started to experience some strange sounds in my ear. What is it, and what should I do?	Basic
2	How can I help myself when I suffer from tinnitus?	
3	Should I believe in the advertisement of a medicine that treats tinnitus in one week?	
4	How to diagnose tinnitus?	
5	Is there a connection between hearing loss and tinnitus?	
6	What is the difference between objective and subjective tinnitus?	Specialized
7	How the tinnitus is connected to otoacoustic emissions?	
8	Is there a link between tinnitus and the psychological state of the patient?	
9	What are the best questionnaires to evaluate tinnitus?	
10	What is the expected result of auditory brainstem response in the case of acoustic neuroma?	

**Table 2:** Average expert scores for each response provided by ChatGPT at the three sessions (session 1 in August 2023, session 2 in November 2023, and session 3 in March 2024). The last column shows the results of the statistical comparison by rmANOVA. There were no statistically significant differences between sessions. SD—standard deviation.

	Average Expert Score (SD)			Test for Difference, *p*-Value
No of Question	Session 1	Session 2	Session 3	
1	4.2 (1.0)	4.7 (0.5)	4.7 (0.5)	*F*(2,10) = 1.66, *p* = 0.23
2	4.0 (0.9)	4.5 (0.5)	4.3 (0.5)	*F*(2,10) = 3.18, *p* = 0.085
3	5.0 (0.0)	5.0 (0.0)	5.0 (0.0)	–
4	4.5 (0.5)	4.8 (0.4)	4.5 (0.5)	*F*(2,10) = 1.0, *p* = 0.40
5	4.3 (0.8)	4.8 (0.4)	4.7 (0.5)	*F*(2,10) = 1.20, *p* = 0.34
6	4.5 (0.5)	4.5 (0.5)	4.8 (0.4)	*F*(2,10) = 1.0, *p* = 0.40
7	4.0 (0.9)	4.3 (0.5)	4.7 (0.5)	*F*(2,10) = 3.33, *p* = 0.077
8	4.8 (0.4)	5.0 (0.0)	4.8 (0.4)	*F*(2,10) = 1.0, *p* = 0.40
9	4.3 (0.5)	4.7 (0.5)	4.5 (0.5)	*F*(2,10) = 1.67, *p* = 0.24
10	4.5 (0.5)	4.5 (0.5)	4.7 (0.5)	*F*(2,10) = 1.0, *p* = 0.40

**Table 3:** Average scores given by the experts and number of responses rated as 5 (extremely satisfactory) and 4–5 (satisfactory and extremely satisfactory). The last column shows the results of the statistical comparison between sessions by rmANOVA. Significant differences are marked by asterisks. SD—standard deviation.

	Mean (SD)			Test for Difference, *p*-Value
	Session 1	Session 2	Session 3	
Average expert score (max = 5)	4.4 (0.3)	4.7 (0.2)	4.7 (0.2)	*F*(2,18) = 9.07, *p* = 0.0019 *
Average expert score—basic (max = 5)	4.4 (0.4)	4.8 (0.2)	4.6 (0.2)	*F*(2,8) = 8.86, *p* = 0.0094 *
Average expert score—specialized (max = 5)	4.4 (0.3)	4.6 (0.2)	4.7 (0.1)	*F*(2,8) = 3.5, *p* = 0.81
Average number of responses rated as 5 (max = 10)	5.3 (2.3)	6.8 (2.4)	6.7 (2.6)	*F*(2,10) = 1.69, *p* = 0.23
Average number of responses rated as 4 or 5 (max = 10)	8.8 (1.5)	10 (0.0)	10 (0.0)	*F*(2,10) = 3.77, *p* = 0.060

**Table 4:** Number of ChatGPT responses that provided sources of information, a number that suggested the help of a specialist, and the average number of words in a response at the three sessions. Results of the statistical comparison are provided in the last column. Significant differences are marked by asterisks. SD—standard deviation.

	Session 1	Session 2	Session 3	Test for Difference, *p*-Value
Number of responses in which sources were provided (0/1, max = 10)	0	0	0	–
Number of responses in which the help of a specialist was suggested (0/1, max = 10)	8	10	7	*χ*^2^ = 3.36, *p* = 0.18
Average number of words (SD)	431 (46)	411 (40)	368 (51)	*F*(2,18) = 22.1, *p* < 0.001 *

## Data Availability

All data underlying this article are attached as a Supplementary File.

## Supplementary Materials

The following supporting information can be downloaded at: https://www.mdpi.com/article/10.3390/brainsci14050465/s1.
