# Supplementary material for: ChatGPT for Tinnitus Information and Support: Response Accuracy and Retest after Three and Six Months
Source: Brain Sci. 2024 May 7;14(5):465. doi: 10.3390/brainsci14050465 (PMC11118795; doi:10.3390/brainsci14050465)
Supplement: Supplementary file 1 [file brainsci-14-00465-s001.zip › Supplementary File S3.pdf]

Responses by ChatGPT – 01.03.2024

1.

I started to experience some strange sounds in the ear? What is it and what should I do?

ChatGPT

Experiencing strange sounds in the ear, such as ringing, buzzing, hissing, or other noises, is often referred to as tinnitus. Tinnitus can vary in severity and may be temporary or persistent. While it is not a condition itself but rather a symptom of an underlying issue, it can be distressing and affect your quality of life.

Here are some common causes of tinnitus:

1. **Exposure to loud noise:** Prolonged exposure to loud noises, such as loud music, machinery, or explosions, can damage the delicate hair cells in the inner ear, leading to tinnitus.
2. **Age-related hearing loss:** As people age, they may experience a decline in hearing ability, which can sometimes be accompanied by tinnitus.
3. **Earwax buildup:** Excessive earwax can block the ear canal and cause tinnitus or temporary hearing loss.
4. **Ear infections or injuries:** Infections or injuries to the ear, such as middle ear infections or perforated eardrums, can cause tinnitus.
5. **Medical conditions:** Certain medical conditions, such as Meniere's disease, temporomandibular joint (TMJ) disorders, or cardiovascular problems, may be associated with tinnitus.

If you are experiencing strange sounds in your ear, it is important to consult with a healthcare professional, preferably an otolaryngologist (ear, nose, and throat specialist), for a proper evaluation and diagnosis. They may conduct a physical examination of your ears, hearing tests, and possibly other diagnostic tests to determine the underlying cause of your tinnitus.

In the meantime, here are some tips that may help manage tinnitus symptoms:

1. **Avoid loud noises:** Protect your ears from loud noises by wearing earplugs or earmuffs when exposed to loud environments.
2. **Manage stress:** Stress and anxiety can exacerbate tinnitus symptoms, so finding ways to manage stress, such as relaxation techniques or therapy, may be helpful.
3. **Maintain a healthy lifestyle:** Eating a balanced diet, getting regular exercise, and avoiding smoking and excessive alcohol consumption can help improve overall health and may alleviate tinnitus symptoms.
4. **Use sound therapy:** White noise machines, fans, or soothing music can help mask the sounds of tinnitus and make them less noticeable, especially when trying to sleep.
5. **Avoid silence:** Complete silence can make tinnitus more noticeable, so having background noise, such as soft music or a gentle fan, can provide relief.

Remember, the best course of action is to seek medical advice from a healthcare professional who can provide personalized recommendations based on your specific situation.

2.

How can I help myself when I suffer from tinnitus?

ChatGPT

If you're experiencing tinnitus, there are several strategies you can try to help manage your symptoms and improve your quality of life. Here are some self-help tips:

1. **Manage stress:** Stress and anxiety can exacerbate tinnitus symptoms. Practice stress-reduction techniques such as deep breathing, meditation, yoga, or progressive muscle relaxation to help calm your mind and body.
2. **Limit exposure to loud noises:** Protect your ears from loud noises by wearing earplugs or earmuffs when you're in environments with loud sounds, such as concerts, sporting events, or construction sites.
3. **Avoid caffeine, nicotine, and alcohol:** Stimulants like caffeine and nicotine can worsen tinnitus symptoms for some people. Similarly, alcohol can dilate blood vessels, which may increase blood flow to the inner ear and exacerbate tinnitus. Limiting or avoiding these substances may help reduce symptoms.
4. **Maintain a healthy lifestyle:** Eating a balanced diet, getting regular exercise, and getting enough sleep can contribute to overall well-being, which may help alleviate tinnitus symptoms.
5. **Use sound therapy:** Background noise can help mask the sounds of tinnitus and make them less noticeable. You can use white noise machines, fans, soothing music, or nature sounds to create a calming environment and distract yourself from the tinnitus.
6. **Try relaxation techniques:** Relaxation techniques such as progressive muscle relaxation, guided imagery, or biofeedback can help you relax and cope with tinnitus-related stress and anxiety.
7. **Cognitive behavioral therapy (CBT):** CBT can help change negative thought patterns and behaviors related to tinnitus, which may help reduce distress and improve coping strategies.
8. **Stay engaged:** Keep yourself engaged in activities you enjoy and spend time with friends and family. Focusing on hobbies, socializing, or volunteering can help take your mind off tinnitus and improve your mood.
9. **Seek support:** Talking to others who understand what you're going through can be helpful. Consider joining a support group for people with tinnitus or connecting with online communities where you can share experiences and coping strategies.
10. **Consult a healthcare professional:** If your tinnitus is persistent or significantly affects your quality of life, consider seeking help from a healthcare professional, preferably an otolaryngologist (ear, nose, and throat specialist) or an audiologist. They can provide personalized recommendations and treatments based on your specific situation.

Remember that what works for one person may not work for another, so it may take some trial and error to find the strategies that work best for you. Be patient and persistent, and don't hesitate to seek professional help if you need it.

3.

Should I believe in the advertisement of a medicine that treats tinnitus in one week?

ChatGPT

It's important to approach advertisements for medications that claim to treat tinnitus in one week with caution and skepticism. Tinnitus is a complex condition with various underlying causes, and there is currently no known cure that works for everyone. While some treatments and management strategies may help alleviate tinnitus symptoms for some individuals, they may not be effective for everyone, and the idea of a quick fix or cure for tinnitus is often unrealistic.

Here are a few points to consider when evaluating advertisements for medications claiming to treat tinnitus in a short period:

1. **Lack of scientific evidence:** Be wary of advertisements that make bold claims without providing scientific evidence or clinical studies to support their claims. Effective treatments for tinnitus are typically backed by research and clinical trials demonstrating their safety and efficacy.
2. **Individual variability:** Tinnitus can have different causes and triggers, and what works for one person may not work for another. It's essential to consider individual variability and the complexity of tinnitus when evaluating treatment options.
3. **Consultation with healthcare professionals:** Before trying any medication or treatment for tinnitus, it's essential to consult with a healthcare professional, preferably an otolaryngologist (ear, nose, and throat specialist) or an audiologist. They can provide a proper evaluation of your condition, determine any underlying causes, and recommend appropriate treatment options based on your specific situation.
4. **Realistic expectations:** While it's natural to seek relief from tinnitus symptoms, it's essential to have realistic expectations about treatment outcomes. Tinnitus management often involves a combination of strategies, including lifestyle changes, sound therapy, counseling, and sometimes medications. Improvement may take time and require patience and perseverance.
5. **Beware of scams:** Unfortunately, there are scams and fraudulent products marketed as "miracle cures" for tinnitus. Be cautious of advertisements that promise unrealistic results or require you to purchase expensive products or treatments without providing credible information or evidence.

In summary, it's advisable to approach advertisements for medications claiming to treat tinnitus in one week with skepticism and to consult with a healthcare professional before trying any new treatment. They can provide guidance and recommendations based on your individual needs and help you make informed decisions about managing your tinnitus.

4.

How to diagnose tinnitus?
ChatGPT

Diagnosing tinnitus typically involves a combination of medical history, physical examination, and hearing tests. Here's an overview of the diagnostic process:

1. **Medical history:** Your healthcare provider will begin by asking you questions about your symptoms, including when you first noticed the tinnitus, the type of sound you hear (e.g., ringing, buzzing, hissing), whether it's constant or intermittent, and if there are any factors that worsen or alleviate the symptoms. They may also inquire about your overall health, medications you're taking, and any previous exposure to loud noises or ear trauma.
2. **Physical examination:** Your healthcare provider will conduct a physical examination, focusing on your ears and auditory system. They may use an otoscope to look for any visible abnormalities in the ear canal or eardrum. They may also check for signs of earwax buildup, inflammation, or other conditions that could contribute to tinnitus.
3. **Hearing tests:** Hearing tests, such as audiometry and tympanometry, may be performed to assess your hearing abilities and identify any underlying hearing loss or abnormalities in the auditory system. These tests can help determine if there are any structural or functional issues in the ear that may be contributing to tinnitus.
4. **Imaging studies:** In some cases, imaging studies such as MRI or CT scans may be ordered to rule out underlying conditions, such as tumors or structural abnormalities in the auditory pathways, that could be causing tinnitus. These tests are typically reserved for individuals with severe or persistent tinnitus or those who have other concerning symptoms.
5. **Specialized tests:** In certain cases, specialized tests may be conducted to further evaluate the underlying cause of tinnitus or assess its impact on your daily life. These tests may include otoacoustic emissions (OAE) testing, auditory brainstem response (ABR) testing, or tinnitus pitch matching tests.

Once a diagnosis of tinnitus is made, your healthcare provider will work with you to develop a treatment plan tailored to your specific needs and symptoms. Treatment options may include sound therapy, counseling, medication, lifestyle changes, or other interventions aimed at managing tinnitus and improving your quality of life. It's essential to work closely with your healthcare provider to address any underlying issues and find effective strategies for managing your tinnitus symptoms.

5.

Is there a connection between hearing loss and tinnitus?

ChatGPT

Yes, there is often a connection between hearing loss and tinnitus. In fact, hearing loss is one of the most common underlying causes of tinnitus. Here's how the relationship between hearing loss and tinnitus works:

1. **Sensorineural hearing loss:** Sensorineural hearing loss occurs when there is damage to the hair cells in the inner ear or the auditory nerve, which impairs the transmission of sound signals to the brain. This type of hearing loss is often associated with aging, exposure to loud noises, or other factors. When the hair cells are damaged, they may send abnormal signals to the brain, which can be perceived as tinnitus.
2. **Compensatory mechanisms:** When the auditory system experiences hearing loss, whether due to aging, noise exposure, or other factors, the brain may try to compensate for the reduced input of sound signals by increasing neural activity. This increased neural activity can manifest as tinnitus, where the brain perceives phantom sounds in the absence of external stimuli.
3. **Correlation between severity:** Research has shown that there is a correlation between the severity of hearing loss and the likelihood and severity of tinnitus. In other words, individuals with more significant hearing loss are more likely to experience tinnitus, and their tinnitus may be more bothersome or intrusive.
4. **Masking effect:** In some cases, hearing aids or other amplification devices used to treat hearing loss can also help alleviate tinnitus. By amplifying external sounds and providing stimulation to the auditory system, hearing aids may help mask the perception of tinnitus and improve overall auditory function.

It's important to note that while hearing loss is a common cause of tinnitus, not everyone with hearing loss will experience tinnitus, and vice versa. Additionally, tinnitus can occur independently of hearing loss and may have other underlying causes, such as ear infections, head or neck injuries, medication side effects, or systemic health conditions.

If you are experiencing tinnitus or hearing loss, it's essential to consult with a healthcare professional, preferably an otolaryngologist (ear, nose, and throat specialist) or an audiologist, for a proper evaluation and diagnosis. They can help determine the underlying cause of your symptoms and recommend appropriate treatment options tailored to your specific needs.

6.

What is the difference between objective and subjective tinnitus?

ChatGPT

The difference between objective and subjective tinnitus lies in the perception of the tinnitus sound and its detectability by others:

1. **Objective tinnitus:**
   - Objective tinnitus refers to tinnitus that can be heard by both the affected individual and others, such as a healthcare provider during an examination. This type of tinnitus is relatively rare and often has an identifiable physical cause, such as blood vessel abnormalities, muscle spasms, or problems with the temporomandibular joint (TMJ). Objective tinnitus can sometimes be heard as a rhythmic pulsing or clicking sound synchronized with the heartbeat or other bodily functions.
2. **Subjective tinnitus:**
   - Subjective tinnitus is the most common type of tinnitus and refers to tinnitus that is only heard by the affected individual and cannot be detected by others. This type of tinnitus is typically caused by problems within the auditory system, such as damage to the hair cells in the inner ear, nerve damage, or changes in neural activity in the auditory pathways. Subjective tinnitus can manifest as various sounds, including ringing, buzzing, hissing, humming, or roaring, and may be constant or intermittent.

In summary, the main difference between objective and subjective tinnitus is whether the tinnitus sound can be heard by others (objective) or is only perceived by the affected individual (subjective). Objective tinnitus is relatively rare and often has an identifiable physical cause, while subjective tinnitus is more common and typically associated with problems within the auditory system.

7.

How the tinnitus is connected to otoacoustic emissions?

ChatGPT

Tinnitus and otoacoustic emissions (OAEs) are connected through their association with the function of the inner ear and the auditory system. Here's how they are related:

1. **Otoacoustic emissions (OAEs):** OAEs are sounds that are generated by the inner ear in response to external auditory stimulation. These sounds are produced by the vibration of the hair cells in the cochlea in response to sound stimuli. OAEs can be measured using a sensitive microphone placed in the ear canal, and they provide valuable information about the function of the outer hair cells in the cochlea.
2. **Tinnitus:** Tinnitus is the perception of sound, such as ringing, buzzing, or hissing, in the absence of any external stimulus. While the exact mechanisms underlying tinnitus are not fully understood, it is believed to involve changes in neural activity in the auditory pathways, including the cochlea, auditory nerve, and auditory cortex in the brain. Tinnitus can be subjective, meaning it is only heard by the affected individual, or objective, meaning it can be heard by others.

The connection between tinnitus and OAEs lies in their shared association with the function of the inner ear, particularly the outer hair cells in the cochlea:

- **Outer hair cell function:** OAEs are a direct measure of the function of the outer hair cells in the cochlea. These hair cells are responsible for amplifying sound signals and enhancing the sensitivity and selectivity of the auditory system. Dysfunction or damage to the outer hair cells can result in abnormalities in OAE measurements, such as reduced or absent OAEs.
- **Tinnitus and outer hair cells:** Changes in the function or integrity of the outer hair cells in the cochlea have been implicated in the development of tinnitus. Damage to the outer hair cells, whether due to noise exposure, aging, or other factors, can lead to alterations in the neural activity in the auditory pathways, potentially contributing to the perception of tinnitus.
- **Clinical implications:** While OAE testing is not typically used as a diagnostic tool for tinnitus, abnormalities in OAE measurements, such as reduced or absent OAEs, may be observed in individuals with tinnitus, particularly those with underlying cochlear dysfunction. OAE testing may be used in conjunction with other diagnostic tests to assess the function of the outer hair cells and identify potential contributing factors to tinnitus.

In summary, while OAEs and tinnitus are distinct phenomena, they are both connected through their association with the function of the inner ear, particularly the outer hair cells in the cochlea. Abnormalities in OAE measurements may provide insights into the underlying cochlear function in individuals with tinnitus.

8.

Is there link between tinnitus and psychological state of the patient?

ChatGPT

Yes, there is a well-established link between tinnitus and the psychological state of the patient. Tinnitus can have a significant impact on an individual's emotional well-being, mental health, and overall quality of life. Here are some ways in which tinnitus and psychological factors are interconnected:

1. **Stress and anxiety:** Tinnitus can cause feelings of stress, anxiety, and worry, particularly when the sound is persistent or bothersome. The constant perception of sound in the absence of external stimuli can be distressing and lead to heightened emotional responses. Conversely, stress and anxiety can also exacerbate tinnitus symptoms, creating a cycle of increased distress and tinnitus perception.
2. **Depression:** Chronic tinnitus can contribute to feelings of sadness, hopelessness, and depression in some individuals. The negative impact of tinnitus on daily life, sleep disturbances, and difficulty concentrating can all contribute to the development of depressive symptoms. Conversely, individuals with pre-existing depression may be more susceptible to the effects of tinnitus on their mental health.
3. **Sleep disturbances:** Tinnitus can interfere with sleep quality and quantity, leading to sleep disturbances such as difficulty falling asleep, frequent awakenings, or early morning awakening. Sleep disturbances can further exacerbate stress, anxiety, and depression, creating a cycle of worsening symptoms.
4. **Social isolation:** Tinnitus can lead to feelings of social isolation and withdrawal from social activities, particularly in noisy environments where tinnitus may be more noticeable. The fear of embarrassment or not being able to hear well in social settings can contribute to avoidance behaviors and social withdrawal, further impacting mental health and well-being.
5. **Coping strategies:** The way individuals cope with tinnitus can also influence their psychological state. Positive coping strategies, such as relaxation techniques, mindfulness practices, or engaging in enjoyable activities, can help reduce stress and improve overall well-being. Conversely, negative coping strategies, such as avoidance behaviors or substance use, can exacerbate tinnitus-related distress and mental health symptoms.

It's essential to recognize the psychological impact of tinnitus and address both the physical and emotional aspects of the condition. Seeking support from healthcare professionals, such as audiologists, psychologists, or counselors, can help individuals develop coping strategies, manage stress and anxiety, and improve their overall quality of life despite living with tinnitus. Additionally, support groups and online communities can provide valuable peer support and resources for individuals affected by tinnitus.

9.

What are the best questionnaires to evaluate tinnitus?

ChatGPT

There are several questionnaires commonly used to evaluate tinnitus severity and its impact on an individual's life. These questionnaires assess various aspects of tinnitus, including its perceived loudness, annoyance, emotional distress, and the extent to which it interferes with daily activities. Here are some of the most commonly used tinnitus questionnaires:

1. **Tinnitus Handicap Inventory (THI):** The THI is one of the most widely used questionnaires for assessing the impact of tinnitus on daily life. It consists of 25 questions that measure the functional, emotional, and catastrophic effects of tinnitus on an individual's quality of life. The THI provides a total score, with higher scores indicating greater tinnitus-related handicap.
2. **Tinnitus Functional Index (TFI):** The TFI is a comprehensive questionnaire that assesses the functional, emotional, and catastrophic effects of tinnitus on an individual's life. It consists of 25 questions across eight subscales, including intrusiveness, sense of control, cognitive interference, sleep disturbance, auditory difficulties, relaxation, quality of life, and emotional distress.
3. **Tinnitus Questionnaire (TQ):** The TQ is a widely used questionnaire that assesses the severity of tinnitus symptoms and their impact on daily life. It consists of 52 questions across three subscales: emotional distress, cognitive distress, and intrusiveness of tinnitus. The TQ provides a total score, with higher scores indicating greater tinnitus-related distress.
4. **Tinnitus Severity Scale (TSS):** The TSS is a simple questionnaire that assesses the severity of tinnitus symptoms based on perceived loudness and annoyance. It consists of two questions that ask individuals to rate the loudness and annoyance of their tinnitus on a scale from 0 to 10.
5. **Tinnitus Reaction Questionnaire (TRQ):** The TRQ is a questionnaire that assesses the emotional distress and psychological impact of tinnitus on an individual's life. It consists of 26 questions across three subscales: emotional distress, cognitive distress, and behavioral reactions to tinnitus.

These questionnaires can be valuable tools for clinicians and researchers to assess tinnitus severity, monitor changes over time, and evaluate the effectiveness of tinnitus treatments. It's essential to select the most appropriate questionnaire based on the specific goals of the assessment and the population being evaluated. Additionally, questionnaires should be used in conjunction with clinical evaluation and other diagnostic tests to obtain a comprehensive understanding of the individual's tinnitus experience.

10.

What is the expected result of auditory brainstem response in case of acoustic neuroma?

ChatGPT

In the case of an acoustic neuroma, also known as vestibular schwannoma, the expected result of auditory brainstem response (ABR) testing may vary depending on several factors, including the size and location of the tumor, its effect on the auditory nerve, and the stage of the disease. Here are some possible outcomes of ABR testing in individuals with acoustic neuroma:

1. **Normal ABR:** In some cases, particularly in the early stages of acoustic neuroma or when the tumor is small and not compressing the auditory nerve, ABR testing may yield normal results. This means that the electrical responses from the auditory nerve and brainstem to auditory stimuli are within normal limits.
2. **Delayed or absent waveforms:** As an acoustic neuroma grows and compresses the auditory nerve or interferes with the transmission of auditory signals, ABR testing may reveal delayed or absent waveforms. This indicates that the electrical responses from the auditory nerve and brainstem are abnormal or attenuated, suggesting dysfunction or impairment of the auditory pathway.
3. **Asymmetric responses:** ABR testing may reveal asymmetric responses between the ears, with one ear showing normal or near-normal responses and the other ear showing delayed or absent waveforms. This asymmetry is often indicative of unilateral hearing loss or asymmetric involvement of the auditory nerve, which can be a red flag for the presence of an acoustic neuroma.
4. **Other abnormalities:** In addition to changes in waveforms, ABR testing may reveal other abnormalities, such as reduced wave amplitudes, prolonged interpeak latencies, or altered morphology of the waveforms. These abnormalities can provide further evidence of dysfunction or pathology along the auditory pathway, including the presence of an acoustic neuroma.

It's important to note that ABR testing is just one component of the diagnostic workup for acoustic neuroma and should be interpreted in conjunction with other clinical findings, imaging studies (such as MRI or CT scans), and patient history. A normal ABR does not necessarily rule out the presence of an acoustic neuroma, particularly in cases of small or early-stage tumors. Conversely, abnormal ABR findings may prompt further evaluation and imaging studies to confirm the diagnosis and determine the extent of the tumor.
